# AHCC inhibited hepatic stellate cells activation by regulation of cytoglobin induction via TLR2-SAPK/JNK pathway and collagen production via TLR4-NF-κβ pathway

**DOI:** 10.1152/ajpgi.00134.2024

**Published:** 2024-09-24

**Authors:** Hayato Urushima, Tsutomu Matsubara, Gu Qiongya, Atsuko Daikoku, Misako Takayama, Chiho Kadono, Hikaru Nakai, Yukinobu Ikeya, Hideto Yuasa, Kazuo Ikeda

**Affiliations:** ^1^Department of Anatomy and Regenerative Biology, Graduate School of Medicine, Osaka Metropolitan University, Osaka, Japan; ^2^Faculty of Pharmacy, Daiichi University of Pharmacy, Fukuoka, Japan; ^3^Laboratory Animal Facility, Graduate School of Medicine, Osaka Metropolitan University, Osaka, Japan

**Keywords:** cytoglobin, fibrosis, hepatic stellate cells, Lentinula edodes mycelia, Toll-like receptor 2 and 4

## Abstract

Cirrhosis, which represents the end stage of liver fibrosis, remains a life-threatening condition without effective treatment. Therefore, prevention of the progression of liver fibrosis through lifestyle habits such as diet and exercise is crucial. The functional food AHCC, a standardized extract of cultured Lentinula edodes mycelia produced by Amino Up Co., Ltd. (Sapporo, Japan)] has been reported to be effective in improving the pathophysiology of various liver diseases. In this study, the aim was to analyze the influence of AHCC on hepatic stellate cells, which are responsible for liver fibrosis. Eight-week-old male C57BL6/j mice were induced with liver fibrosis by intraperitoneal injection of carbon tetrachloride. Simultaneously, they were orally administered 3% AHCC to investigate its impact on the progression of liver fibrosis. Using the human hepatic stellate cell (HHSteC) line, we analyzed the influence of AHCC on the expression of molecules related to hepatic stellate cell activation. The administration of AHCC resulted in reduced expression of collagen1a, α smooth muscle actin (αSMA), and heat shock protein 47 in the liver. Furthermore, the expression of cytoglobin, a marker for quiescent hepatic stellate cells, was enhanced. In vitro study, it was confirmed that AHCC inhibited αSMA by inducing cytoglobin via upregulating the stress-activated protein kinase/Jun NH_2_-terminal kinase (SAPK/JNK) pathway through Toll-like receptor (TLR) 2. In addition, AHCC suppressed collagen1a production by hepatic stellate cells through TLR4-NF-κβ pathway. AHCC was suggested to suppress hepatic fibrosis by inhibition of hepatic stellate cells activation. Daily intake of AHCC from mild fibrotic stages may have the potential to prevent the progression of liver fibrosis.

**NEW & NOTEWORTHY** AHCC, a standardized extract of cultured *Lentinula edodes* mycelia, suppresses liver fibrosis progression by induction of cytoglobin via the Toll-like receptor 2 (TLR2)-stress-activated protein kinase/Jun NH_2_-terminal kinase (SAPK/JNK) pathway and the inhibition of collagen production via the TLR4-NFκβ pathway in hepatic stellate cells. Daily oral administration of AHCC from the stage of MASLD may have the potential to prevent disease progression to MASH with fibrosis.

## Graphical abstract

**Figure:**
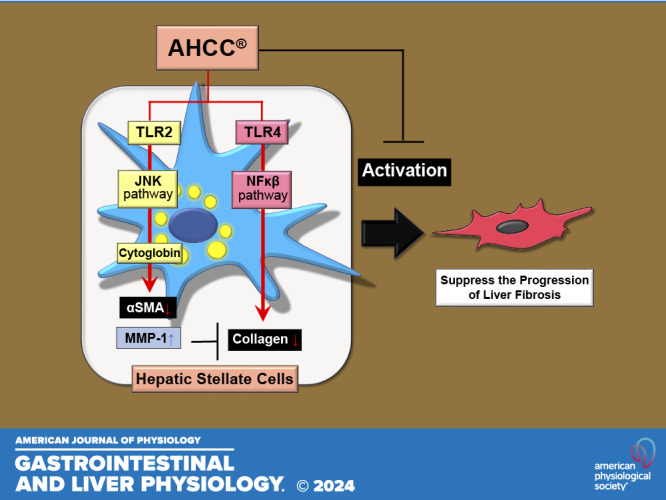

## INTRODUCTION

The space between hepatocytes and sinusoidal endothelial cells is called the Disse space, where hepatic stellate cells (HSCs) are present surrounding the sinusoidal endothelial cells (1). In a normal liver, HSCs exist as an inactive form and store vitamin A (2). Meanwhile, during liver damage, HSCs are activated by various factors originating from hepatocytes, Kupffer cells, sinusoidal endothelial cells, and other sources. Upon activation, they transform into contractile myofibroblasts with the ability to secrete extracellular matrix components such as collagen (3). In transient liver damage, the extracellular matrix secreted by activated HSCs serves as a scaffold for the regeneration of hepatocytes. After inflammation subsides, these extracellular matrixes are rapidly degraded by collagenases like matrix metalloproteinase-1 (MMP-1). However, the constitutive activation of HSCs in chronic liver disease leads to pathological liver fibrosis due to the excessive accumulation of extracellular matrix in the Disse space (4). Cirrhosis, the end-stage of liver fibrosis, is not only responsible for liver dysfunction but also represents a critical risk factor for liver cancer, making it a fatal condition (5). Therefore, preventing the progression of liver fibrosis through the inhibition of HSCs activation is crucial. However, thus far, no promising agents for preventing the progression of liver fibrosis have been identified. Generally, liver fibrosis progresses over several years. Particularly in recent years, steatohepatitis progressing from hepatic steatosis due to lipid metabolism dysfunction in hepatocytes, especially caused by high-fat diets and lack of exercise, has emerged as a significant cause of liver fibrosis (6, 7). Therefore, improving daily lifestyle habits such as diet and exercise is crucial for preventing the progression of liver fibrosis (8, 9).

Active hexose correlated compound (AHCC) is a standardized extract of cultured *Lentinula edodes* mycelia, produced by Amino Up Co., Ltd. (Sapporo, Japan) (10). AHCC has been reported to have immunomodulatory effects such as increase of dendritic cell in peripheral blood (11), promotion of clearance of infected papillomavirus (12), and enhancement of antibody titers after influenza vaccination (13). In the context of liver pathogenesis, clinical trial revealed that administering AHCC after liver cancer resection improved the prevention of cancer recurrence and overall survival (14, 15). Importantly, the group receiving AHCC orally showed a significantly lower prevalence of liver cirrhosis compared with the nonintake group (14). Therefore, it is suggested that AHCC may have some influence on the progression of liver fibrosis. However, the preventive effects of AHCC on the progression of liver fibrosis, with a focus on its impact on HSCs activation, have not yet been clearly elucidated.

In this study, we verified whether AHCC intake suppresses the progression of liver fibrosis by the suppression of HSCs activation using a carbon tetrachloride-induced mouse fibrosis model and revealed that AHCC increased the expression of cytoglobin. Since cytoglobin has been reported to have antifibrotic effect through the suppression of HSCs activation (16), we sought the mechanism of enhancement of cytoglobin by AHCC. Here we show for the first time that AHCC suppresses liver fibrosis progression by induction of cytoglobin via the Toll-like receptor 2 (TLR2)-stress-activated protein kinase/Jun NH_2_-terminal kinase (SAPK/JNK) pathway and inhibition of collagen production via the TLR4-NF-κβ pathway in hepatic stellate cells.

## MATERIALS AND METHODS

### Animal Model

For liver fibrosis model, 8-wk-old male C57BL/c mice were given distilled water (control group) or 3% AHCC (ad libitum, Amino Up Co., Ltd, Hokkaido, Japan, AHCC group; *n* = 6 each). We replaced the distilled water or AHCC water twice a week. Liver fibrosis was induced by 16 times intraperitoneal injections of carbon tetrachloride (CCl_4_; twice a week, 0.5 µL/g of body weight, dissolved in corn oil at a ratio of 1:3). A day after final CCl_4_ administration, serum and liver tissue were sampled. For the comparison of cytoglobin expression in liver, a single-dose CCl_4_ administration model was used. Eight-week-old male C57BL/c mice were given distilled water (vehicle group) or 3% AHCC (AHCC group) for 7 days. Then, CCl_4_ (0.5 µL/g of body weight, dissolved in corn oil at a ratio of 1:3) was intraperitoneally injected. Liver tissue was sampled 24 h after CCl_4_ injection. These animal experiments were approved by the institutional animal care and use committee of Osaka Metropolitan University (No. 18061) and was conducted in compliance with the ARRIVE guidelines (17).

### In Vitro Study

The human HSCs line HHSteCs (Lot No. 10326) was purchased from ScienCell Research Laboratories (Carlsbad, CA). These cells were maintained using the Stellate Cell Medium set (Cat. No. 5301) at 37°C in a humidified 5% CO_2_ atmosphere. HHsteC cells were seeded in a 12-well plate at a density of 1.0 × 105 cells per well and cultured in DMEM medium containing 10% FBS. The following day, the medium was replaced with AHCC-adjusted DMEM medium at concentrations of 100 or 500 μg/mL. Sampling was conducted 48 h after the addition of AHCC. The concentration of AHCC was determined based on the previous studies (18). For signaling pathway analysis, HHSteCs were treated with or without AHCC (500 μg/mL) in the presence or absence of TLR2 or TLR4 inhibitors. Then, HHSteCs were harvested at indicated times in Figs. 4 and 5. In transient transfection assays, HHSteCs were transfected using Lipofectamine RNAiMAX (Thermo Fisher Scientific, Waltham, MA) for siRNA transfection of c-Jun (JUNVHS40918, Invitrogen), for cytoglobin (Thermo Fisher Scientific), or RelA (NF-κβ p65) (Thermo Fisher Scientific). TLR2 ligand, zymosan, was from Novus Biologicals (Littleton, CO). The inhibitor experiment was conducted using the following chemicals. JNK Inhibitor, SP600125 (5 or 50 nM), was from Fujifilm Co. (Tokyo, Japan). TLR2 inhibitor, TLR2-IN-C29 (50 μM) and TLR4 inhibitor, TLR4-IN-C34 (5 μM) were from Selleck Chemicals (Houston, TX).

### Primary Antibodies

The following antibodies were used for Western blot (WB) analysis, immunohistochemistry (IHC), and immunofluorescence (IF): anti-collagen1A (E8F42, WB; 1:2,000, IHC; 1:200), anti-SAPK/JNK (No. 9252, WB; 1:2,000), antiphosphorylated SAPK/JNK (81E11, WB; 1:2,000), anti-c-Jun (60A8, WB; 1:2,000), antiphosphorylated c-Jun (54B3, WB; 1:2,000), anti-NF-κβ p65 (D14E12, WB; 1:2,000), antiphosphorylated-NF-κβ p65 (S468, WB; 1:1,000), and caspase-3 (No. 9662, IHC; 1:200) were from Cell Signaling Technology (Danvers, MA), and anti-αSMA (1A4, WB; 1:5,000, IHC; 1:2,000) was from DAKO. Anti-desmin (goat polyclonal, IHC; 1:200) was from Invitrogen (Carlsbad, CA). Anti-HSP47 (G-12, WB; 1:5,000, IHC; 1:1,000) was from Santa Cruz Biotechnology (Santa Cruz, CA). Anti-cytoglobin (HPA017757, WB; 1:2,000, IHC and IF; 1:200) was from Sigma-Aldrich (St. Louis, MO) or kindly gifted by Prof. Norifumi Kawada (Osaka Metropolitan University). Anti-GAPDH (MAB-374, WB; 1:5,000) was from Millipore (Billerica, MA), and 8-OhdG was from Nikken Zail (Shizuoka, Japan, IHC; 1:200).

### Quantitative PCR of Analysis

RNA was extracted from cells using TRIzol reagent (Thermo Fisher Scientific) and Direct-zol RNA Miniprep (Zymo Research, Irvine, CA). Quantitative PCR (qPCR) was performed using cDNA generated from RNA and the SuperScript III Reverse Transcriptase kit (Thermo Fisher Scientific). qPCR reaction was carried out using SYBR green PCR master mix (Thermo Fisher Scientific) in the Thermal Cycler Dice Real Time System 2 (TAKARA BIO). The values were quantified using the comparative CT method and were normalized to 18S ribosomal RNA. The data were expressed as the ratio to the average of the control or vehicle group. The primers used in this study are listed in Table 1.

**Table 1.** Primer list

Mouse Gene Names	Primer Sequences
*Tgfb1*	
Forward	5′-TCGAGGGCGAGAGAAGTTTA-3′
Reverse	5′-AAAAGAATGTCCCGGCTCTC-3′
*Tnfa*	
Forward	5′-TCCCAGGTTCTCTTCAAGGGA-3′
Reverse	5′-GGTGAGGAGCACGTAGTCGG-3′
*Il1b*	
Forward	5′-TTGACGGACCCCAAAAGATG-3′
Reverse	5′-TGGACAGCCCAGGTCAAAG-3′
*Acta2*	
Forward	5′-CGAAACCACCTATAACAGCATCA-3′
Reverse	5′-GCGTTCTGGAGGGGCAAT-3′
*Col1a1*	
Forward	5′-CCAAGGGTAACAGCGGTGAA-3′
Reverse	5′-CCTCGTTTTCCTTCTTCTCCG-3′
*SERPINH1*	
Forward	5′-AGGTCACCAAGGATGTGGAG-3′
Reverse	5′-CAGCTTCTCCTTCTCGTCGT-3′
*18S*	
Forward	5′-CGGCTACCACATCCAAGGAA-3′
Reverse	5′-ATTGGAGCTGGAATTACCGC-3′

Human Gene Names	Primer Sequences
*ACTA2*	
Forward	5′-GACCGAATGCAGAAGGAGAT-3′
Reverse	5′-CACCGATCCAGACAGAGTATTT-3′
*COL1A1*	
Forward	5′-AAGAGGAAGGCCAAGTCGAG-3′
Reverse	5′-CACACGTCTCGGTCATGGTA-3′
*COL1A2*	
Forward	5′-GAAAAGGAGTTGGACTTGGC-3′
Reverse	5′-AGCAGGTCCTTGGAAACCTT-3′
*SERPINH1*	
Forward	5′-AGAGTAGAATCGTGTCGCGG-3′
Reverse	5′-CTGAGAAGCAGGAGGGAGC-3′
*MMP1*	
Forward	5′-CAGAGATGAAGTCCGGTTTTTC-3′
Reverse	5′-GGGGTATCCGTGTAGCACAT-3′
*CYGB*	
Forward	5′-CGAGATGGAGATCGAGCG-3′
Reverse	5′-CGAGGGGAAGTTCACAAAGA-3′
*18S*	
Forward	5′-CAGCCACCCGAGATTGAGCA-3′
Reverse	5′-TAGTAGCGACGGGCGGTGTG-3′

Data are expressed as means ± SD of three different samples.

### Western Blot Analysis

Cells were homogenized with RIPA buffer (50 mM Tris·HCl at pH 7.5, 150 mM NaCl, 1% Triton X-100, 1% SDS) containing protease inhibitor cocktail cOmplete Mini (Roche, Basel, Switzerland) and phosphatase inhibitors (1 mM sodium fluoride, 1 mM β-glycerol phosphate, and 1 mM sodium vanadate). Protein samples were subjected to 8–15% SDS-polyacrylamide gel electrophoresis and were transferred to polyvinylidene difluoride membranes using standard Western blot techniques. After being blocked with 5% skim milk, the membranes were probed with primary antibodies diluted at 1:1,000 to 5,000, and horseradish peroxidase-conjugated secondary antibodies were diluted at 1:5,000. Immunoreactive bands were visualized using the ImmunoStar Zeta or ImmunoStar LD system and were detected using the LAS3000 or LAS4000 device (GE Healthcare, Chicago, IL). WB Stripping Solution (Nacalai Tesque, Kyoto, Japan) was used to remove the antibodies from the Western blot membrane for reprobing. The quantification of Western blot bands was performed by using ImageJ software v. 1.52 (NIH, Bethesda, MD) and normalized to GAPDH or control. Data are expressed as means ± SD of three different experiments.

### Flow Cytometry

HHSteC cells were stained with anticytoglobin or isotype control (RTK2071, BioLegend, San Diego, CA) followed by staining with Alexa Fluor 488 anti-rabbit antibody (Invitrogen). Then the expression of cytoglobin was characterized using DxFlex cytometer (Beckman Coulter, Brea, CA).

### Histological, Immunohistochemical, and Immunofluorescence Analysis

For histological evaluation, 4% paraformaldehyde fixative, fixed in 0.1 M phosphate buffer-fixed, paraffin-embedded mouse liver sections were stained with hematoxylin-eosin (HE). For immunohistochemical analysis, they were deparaffinized, hydrated, heated to 110°C in citrate buffer for 20 min, depleted of endogenous peroxidase activity, and then blocked with Blocking One (Nacalai Tesque) for 1 h. Next, the slides were treated with primary antibodies overnight at 4°C. The slides were incubated with secondary antibody (Bioss, Inc., Woburn, MA) for 1 h at room temperature. The reaction was visualized by diaminobenzene (DAB) substrate (Vector Laboratories, Burlingame, CA). All specimens were counterstained with hematoxylin. For immunofluorescence analysis, after hybridization with primary antibodies, the slides were incubated with Alexa-fluor 488 or 594 conjugated donkey anti-mouse or rabbit IgG (Invitrogen). DAPI (Dojindo, Kumamoto, Japan) was used as a nuclear marker.

### Statistical Analysis

All data are expressed as the means ± standard deviation. All data were analyzed by using ANOVA with a post hoc Dunnet’s test or unpaired Student’s *t* test. These analyses were performed using JASP (JASP team 2023, v. 0. 18. 1).

## RESULTS

### AHCC Suppressed CCl_4_-Induced Liver Fibrosis

**Figure 1.**
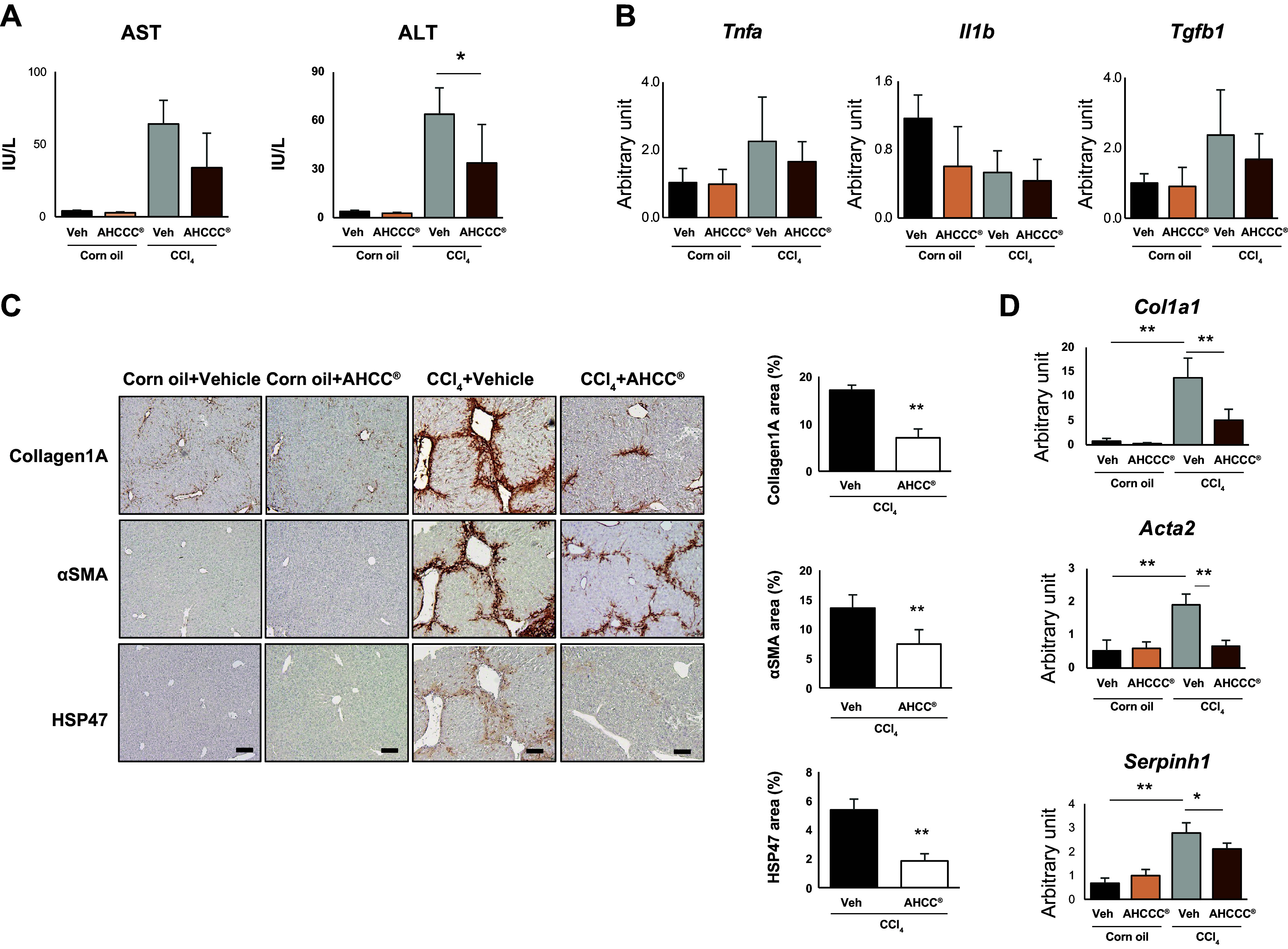
AHCC ameliorates CCl_4_-induced liver fibrosis in a mouse model. Eight-week-old male C57BL/c mice were given distilled water (control group) or 3% AHCC (ad libitum, AHCC group, *n* = 6 each). Liver fibrosis was induced by 16 times intraperitoneal injections of carbon tetrachloride (CCl_4_, twice a week, 0.5 µL/g of body weight, dissolved in corn oil at a ratio of 1:3). A day after final CCl_4_ administration, serum and liver tissue were sampled. AHCC was administered orally throughout the experimental period. *A*: serum levels of ALT and AST. Data are presented as means ± SD. *P < 0.05 (*n* = 6, vs. vehicle CCl_4_ vehicle group). *B*: gene expression related to inflammation in liver. *Tnfa* encoding tumor necrosis factor α. *Il1b* encoding interleukin 1 β. *Tgfb1* encoding transforming growth factor β1. *n* = 6. *C*: representative images of immunohistochemistry for collagen1A (*f left*), αSMA (*middle left*), and HSP47 (*bottom left*). *Right*: quantification of each positive area. **P < 0.01 (*n* = 6, vs vehicle CCl_4_ group). Scale bar; 100 μm. *D*: gene expression related to activated stellate cells in the liver. *Col1a1* encoding collagen1A1. *Acta2* encoding αSMA. *Serpinh1* encoding HSP47. *P < 0.05, *P < 0.01 (*n* = 6, vs. vehicle CCl_4_ group). AHCC, active hexose correlated compound; ALT, alanine aminotransferase; AST, aspartate aminotransferase; CCL_4_, carbon tetrachloride; HSP47, heat shock protein 47.

The serum aspartate aminotransferase (AST) and alanine aminotransferase (ALT) levels, markers of hepatitis, increased due to the administration of CCl_4_, but the intake of AHCC significantly suppressed the elevation in ALT (Fig. 1*A*). On the other hand, the gene expression of inflammatory cytokines *Tnfa*, *Il1b*, and *Tgfb* in liver tissue showed no difference between the vehicle group and the AHCC group (Fig. 1*B*). Immunohistochemical staining revealed that the indicators of liver fibrosis, Collagen1a, as well as the positive areas for activated HSCs markers αSMA and heat shock protein 47 (HSP47) significantly decreased with the intake of AHCC (Fig. 1*C*). Furthermore, the enhanced expression of *Col1a1* (encoding Collagen1A), *Acta2* (encoding αSMA), and *Serpinh1* (encoding HSP47) genes in the liver due to CCl_4_ administration was suppressed by AHCC intake (Fig. 1*D*).

### AHCC Inhibited the Activation of Human HSCs In Vitro

**Figure 2.**
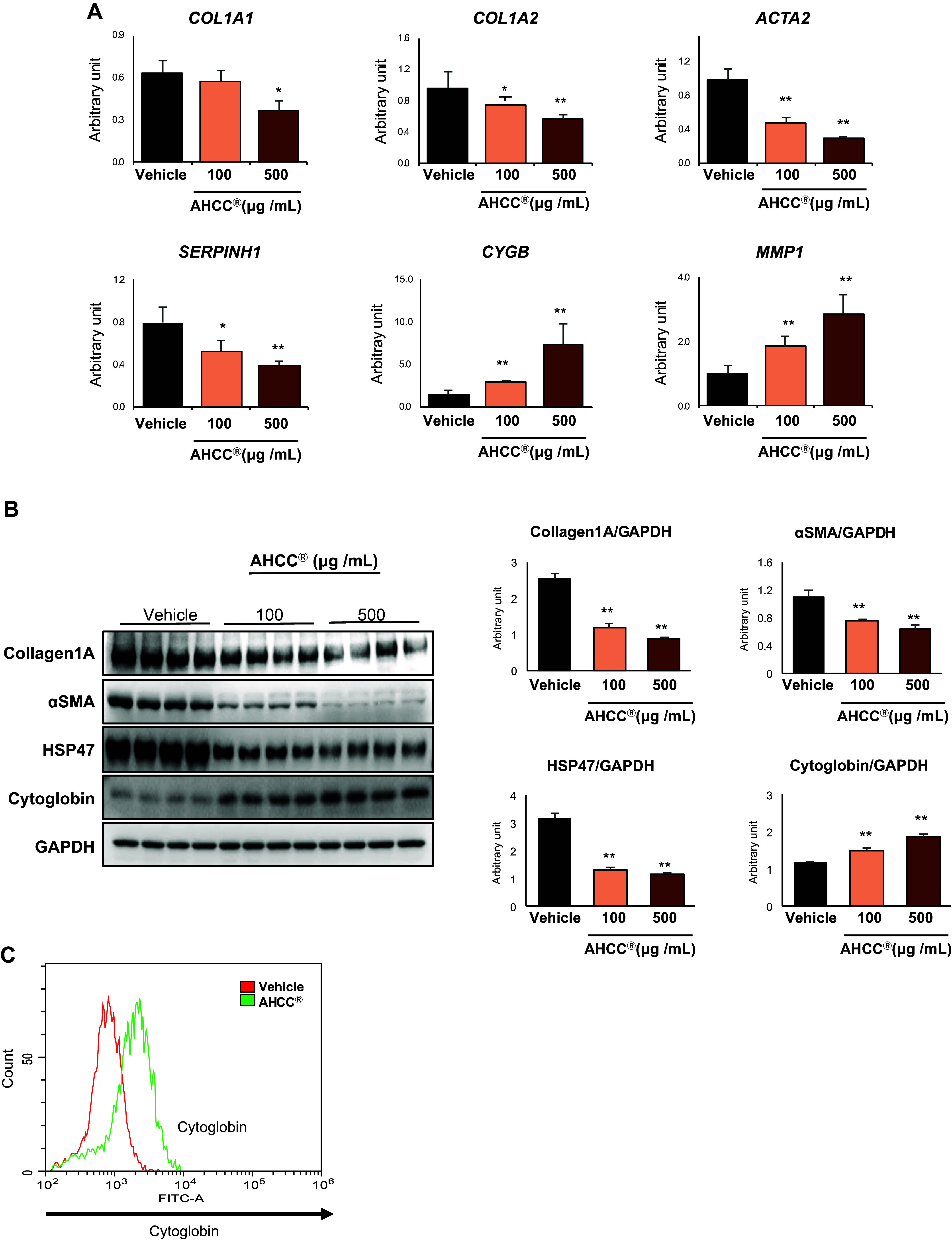
AHCC inhibited the activation of human HSCs in vitro. Human hepatic stellate cells (HHsteC) were treated with or without treatment of AHCC (100 or 500 μg/mL) for 48 h. Gene expression related to activated (*COL1A1*, *COL1A2*, *ACTA2*, and *SERPINH1*) or quiescent (*CYGB* and *MMP1*) hepatic stellate cell are shown in *A*. *CYGB* encoding cytoglobin and *MMP1* encoding matrix metalloproteinase-1, and protein expression is shown in *B* (the qPCR experiment was performed in triplicate, and the WB data are presented from experiments conducted with *n* = 3). *C*: flow cytometry analysis for cytoglobin expression in HHSteC cells. The control group is represented in red, and the AHCC group is represented in green. Data are shown as means ± SD. *P < 0.05 and **P < 0.01 (vs. vehicle). AHCC, active hexose correlated compound; HSCs, hepatic stellate cells; qPCR, quantitative PCR; WB, Western blot.

We investigated the inhibitory effect of AHCC on HSCs activation using the human hepatic stellate cell line, HHSteC, in an in vitro setting. The addition of AHCC significantly suppressed the expression of genes *ACTA2, SERPINH1*, *COL1A1*, and *COL1A2*, which are markers for activated HSCs. Conversely, it enhanced the expression of genes *CYGB* and *MMP*1, markers for quiescent HSCs. (Fig. 2*A*). Western blot analysis revealed that the protein expression of Collagen1a, αSMA, and HSP47 was suppressed, whereas conversely enhancing the protein expression of cytoglobin encoded by *CYGB* (Fig. 2*B*). Furthermore, flow cytometry also showed the increase of cytoglobin expression in HHSteC cells by AHCC stimulation (Fig. 2*C*). Cytoglobin has been reported to exert antifibrotic effects in various liver fibrosis models (19–21). Therefore, we focused on the effect of AHCC on the induction of cytoglobin as a mechanism of the suppression of HSCs activation.

### AHCC Enhanced the Expression of Cytoglobin in HSCs during Liver Injury and Suppressed the Production of Reactive Oxygen Species

**Figure 3.**
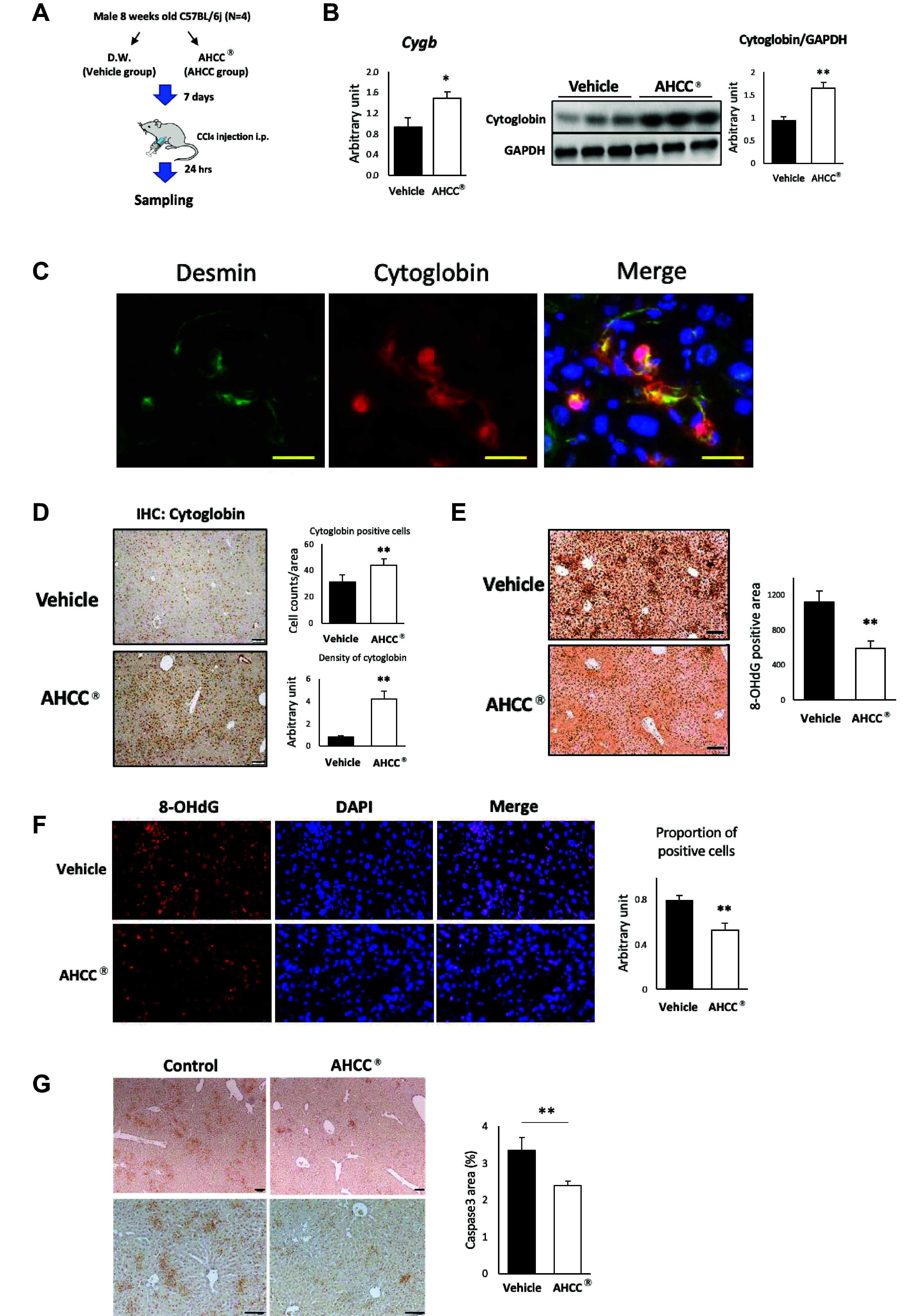
AHCC enhanced the expression of cytoglobin in HSCs during liver injury and suppressed the production of reactive oxygen species. *A*: protocol of a single-dose CCl_4_ administration model in this study. Eight-week-old male C57BL/c mice were given distilled water (vehicle group) or 3% AHCC (AHCC group) for 7 days (*n* = 4). Then, CCl_4_ (0.5 µL/g of body weight, dissolved in corn oil at a ratio of 1:3) was intraperitoneally injected. Liver tissue was sampled 24 h after CCl_4_ injection. *B*: gene (*left*) and protein (*middle*) expression of cytoglobin in liver. *Right*: data quantifying the WB. Data are expressed as means ± SD (the qPCR experiment was performed in triplicate, and the WB data are presented from experiments conducted with *n* = 3). *P < 0.05 and **P < 0.01 (vs. vehicle). *C*: immunofluorescence images for cytoglobin or desmin, a hepatic stellate cell marker. Scale bar; 20 μM. *D*: immunohistochemistry images for cytoglobin in liver (*left*). The quantification of cell counts of cytoglobin positive cells is shown in *right* panel. Scale bar; 100 μm. *Right*: data quantifying the number of cytoglobin-positive cells and the staining intensity of cytoglobin. Immunohistochemical detection (*E*) and immunofluorescence (*F*) for 8-OHdG in liver. DAPI (4′,6-diamidino-2-phenylindole) was used for nuclear staining. The data quantifying the immunohistochemistry are shown in the *right* panel of each. Scale bar in *E*; 100 µm and in *F*; 20 μm. *G*: representative images of immunohistochemistry for caspase-3. *Right*: quantification of positive area. **P < 0.01 (vs. vehicle CCl_4_ group). Scale bar; 100 μm. AHCC, active hexose correlated compound; CCL_4_, carbon tetrachloride; HSCs, hepatic stellate cells; qPCR, quantitative PCR; WB, Western blot.

We verified the induction effect of cytoglobin by AHCC using an in vivo model of single-dose CCl_4_-induced liver injury (Fig. 3*A*). Twenty-four hours after CCl_4_ administration, the expression of the *Cygb* gene and the protein expression of cytoglobin in the liver of AHCC group were significantly increased compared with the vehicle group (Fig. 3*B*). Furthermore, in the fluorescent double staining of HSC markers desmin and cytoglobin, cytoglobin-positive cells coincided with desmin-positive cells (Fig. 3*C*). From this, it was confirmed that cytoglobin is expressed in HSCs. In addition, immunostaining for cytoglobin in liver tissue revealed a significant increase in the number and the density of cytoglobin-positive cells and by the intake of AHCC (Fig. 3*D*). It has been reported that cytoglobin in HSCs prevents oxidative damage to surrounding liver cells by eliminating nitric oxide, a type of reactive oxygen species (ROS) produced during liver impairment (22). We therefore stained 8-OHdG, an indicator of oxidative damage in cells (23). As shown in Fig. 3*E*, in the AHCC intake group, a decrease in the 8-hydroxy-2′-deoxyguanosine (8-OHdG)-positive area was observed compared with the vehicle group. In addition, although in the vehicle group most of the nuclei stained with DAPI overlapped with 8-OHdG, in the AHCC group, numerous nuclei were negative for 8-OHdG. Since it is known that ROS-induced DNA damage causes apoptosis in hepatocytes (24), we next performed caspase-3 immunostaining of liver tissue. As shown in Fig. 3*G*, oral administration of AHCC significantly reduced the caspase-3-positive areas in CCl_4_-induced liver injury. These results suggest that AHCC enhances cytoglobin expression in HSCs, thereby suppressing hepatocyte damage by inhibiting the generation of ROS.

### AHCC Induced Cytoglobin Expression via the SAPK/JNK Signaling Pathway

**Figure 4.**
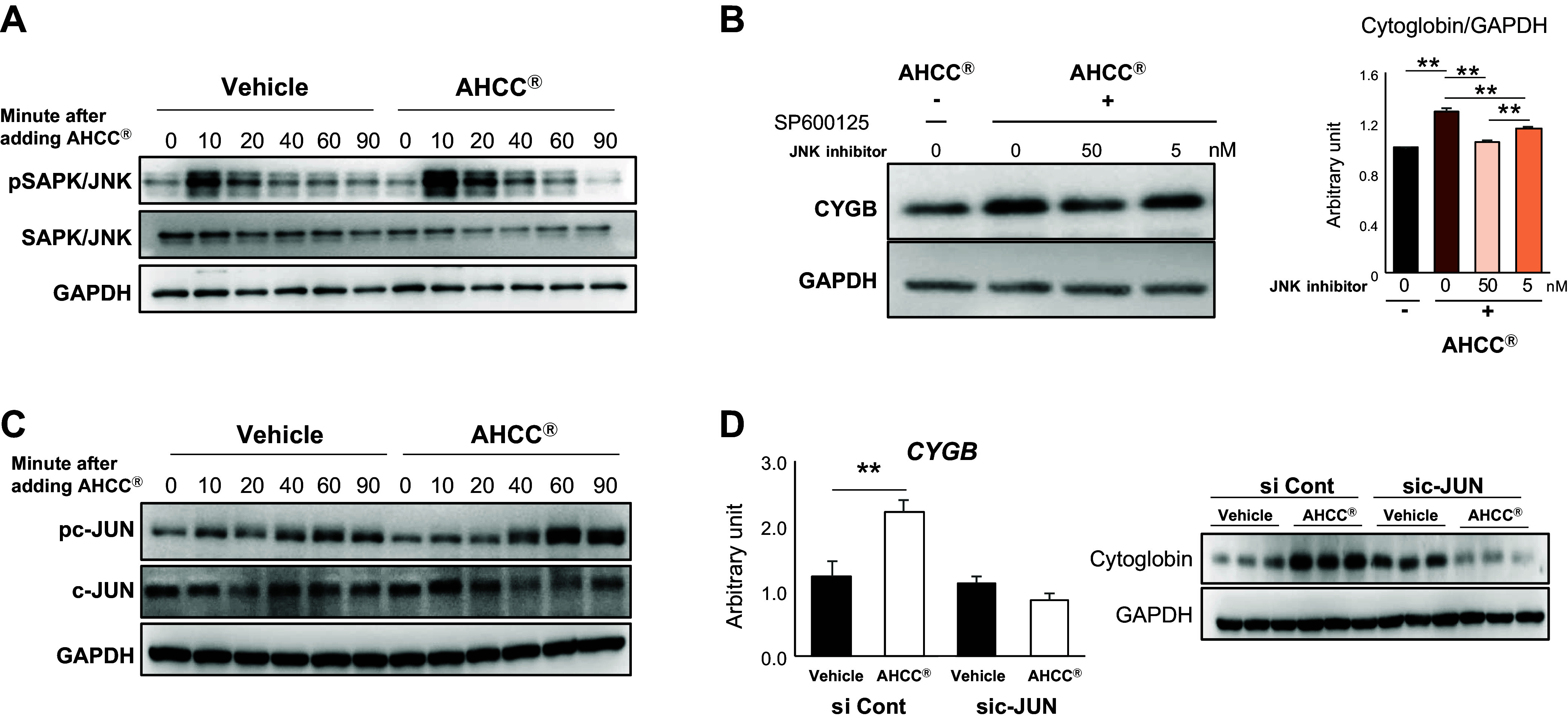
AHCC induced cytoglobin expression via the SAPK/JNK signaling pathway. HHSteC cells were treated with or without AHCC (500 μg/mL) and harvested at the indicated times. *A*: representative Western blotting image for SAPK signaling. *B*: influence of JNK inhibitor on cytoglobin induction by AHCC. *Right*: the quantification of Western blot bands was normalized to GAPDH. *C*: representative Western blotting image for c-JUN signaling, a downstream of SAPK/JNK. *D*: alteration of cytoglobin-inducing capability by AHCC under c-jun knockdown. *Left*: gene expression of cytoglobin. *Right*: cytoglobin protein expression. Data are expressed as means ± SD (experiment was performed in triplicate). **P < 0.01 (vs. vehicle). AHCC, active hexose correlated compound; HHSteC, human hepatic stellate cells; SAPK/JNK; stress-activated protein kinase/Jun NH_2_-terminal kinase.

Next, we analyzed the impact of AHCC on the SAPK/JNK pathway, previously reported as the pathway inducing cytoglobin expression (25) in HSCs. The phosphorylation of SAPK/JNK was enhanced by AHCC, and the induction effect of cytoglobin expression by AHCC was canceled by a SAPK/JNK inhibitor (Fig. 4, *A* and *B*). Furthermore, AHCC enhanced the phosphorylation of c-JUN, downstream of the SAPK/JNK pathway, whereas the enhancement effect of AHCC on the cytoglobin expression was attenuated by JNK gene knockdown (Fig. 4, *C* and *D*). These results revealed that AHCC induced cytoglobin expression via the SAPK/JNK signaling pathway.

### AHCC Increased Cytoglobin Production in HSCs via Toll-Like Receptor 2

**Figure 5.**
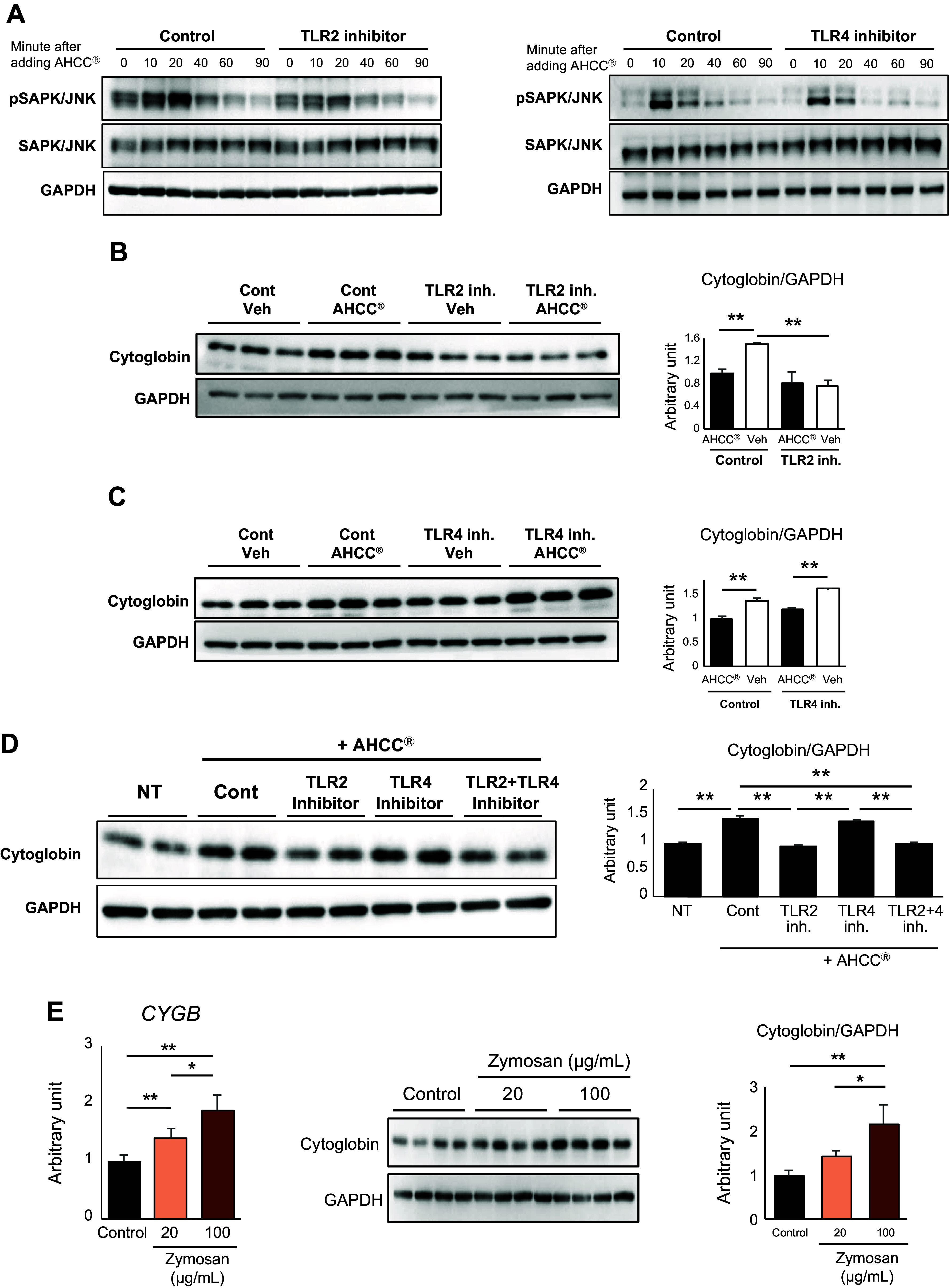
AHCC increased cytoglobin production in HSCs via Toll-like receptor 2. *A–D*: HHSteC cells were treated with AHCC (500 μg/mL) in the presence or absence of TLR2 (50 μM) or TLR4 (5 μM) inhibitor and harvested at the indicated times (*A*) or 48 h after AHCC treatment (*B*, *C*, and *D*). The representative Western blotting images for SAPK/JNK signaling are shown. The quantification of Western blot bands was normalized to GAPDH and shown in *right* panel of each. Data are expressed as means ± SD (WB data are presented from experiments conducted with *n* = 3). **P < 0.01 (vs. vehicle). *E*: HHsteC cells were treated with zymosan (20 or 100 μg/mL) for 48 h. Gene (*left*) and protein (*middle*) of cytoglobin are shown. *Right*: quantification of Western blot bands was normalized to GAPDH. Data are expressed as means ± SD (WB data are presented from experiments conducted with *n* = 4). **P < 0.01, *P < 0.05 (vs. control). AHCC, active hexose correlated compound; Cont, control; HHSteC, human hepatic stellate cells; HSCs, hepatic stellate cells; Inh., inhibitor; NT, no treatment; SAPK/JNK; stress-activated protein kinase/Jun NH_2_-terminal kinase; Veh, vehicle; WB, Western blot.

It has been reported that AHCC stimulates TLR2 and TLR4, which are receptors of the innate immune system, as ligands (26). In addition, given that the SAPK/JNK pathway exists downstream of TLR2 and TLR4 signaling, we then investigated the influence of AHCC on TLR2 and TLR4 pathway. The addition of a TLR2 inhibitor significantly decreased the activation of the SAPK/JNK pathway (Fig. 5*A*, *left*). On the other hand, the addition of a TLR4 inhibitor only slightly attenuated the phosphorylation of the SAPK/JNK (Fig. 5*A*, *right*). The enhancement effect of AHCC on cytoglobin production was weakened by the addition of a TLR2 inhibitor (Fig. 5*B*), whereas there was no significant change with the addition of a TLR4 inhibitor (Fig. 5*C*). Next, we investigated the additive or synergistic effect of inhibiting both TLR2 and TLR4; however, the effect was only equivalent to that of inhibiting TLR2 alone (Fig. 5*D*). Moreover, stimulation of HHSteC cells with zymosan, a TLR2 ligand (27), increased the expression of cytoglobin in a dose-dependent manner (Fig. 5*E*).

### Different Mechanisms of Activated HSCs Marker Regulation by AHCC

**Figure 6.**
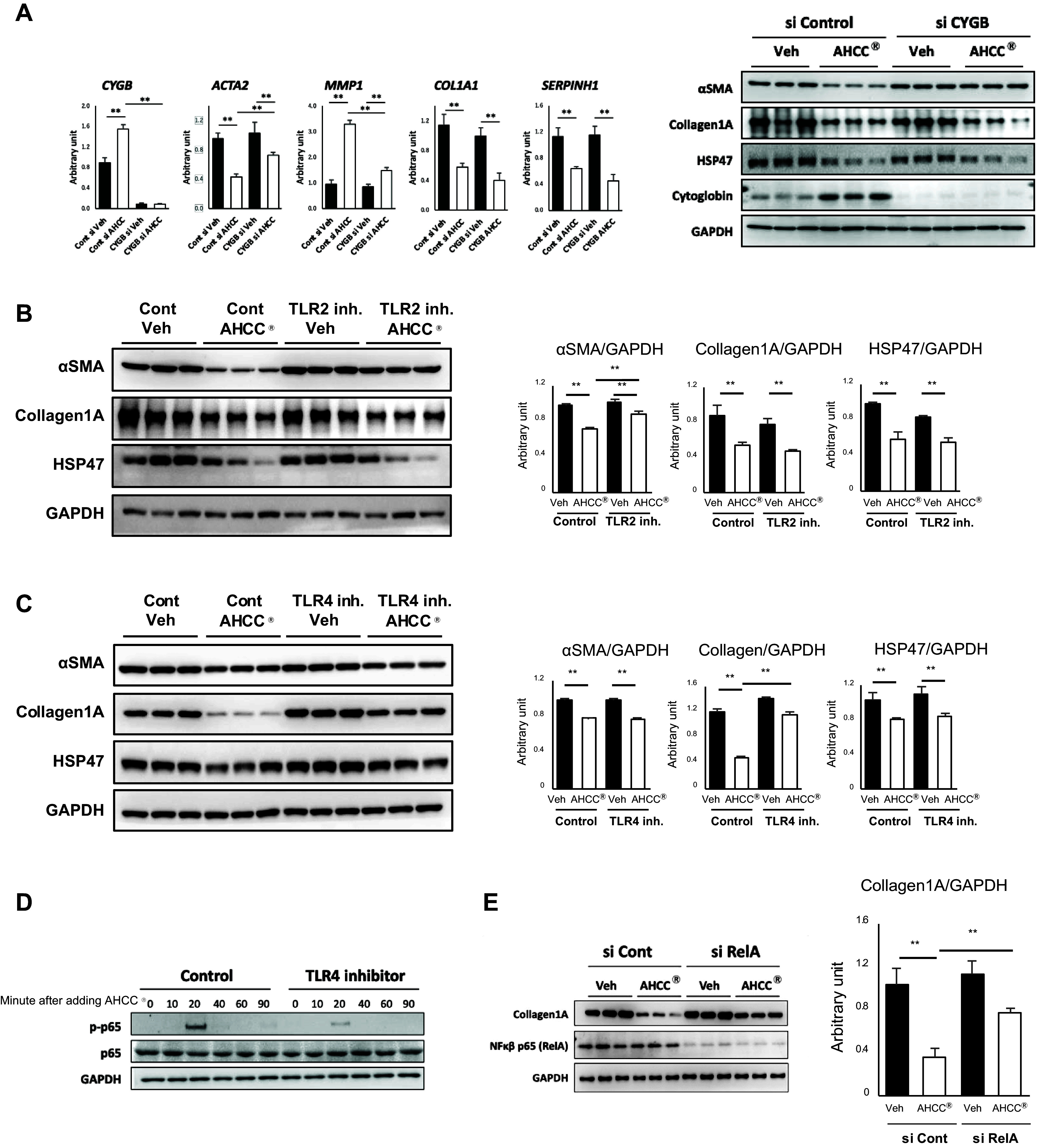
AHCC regulated αSMA expression via TLR2-cytoglobin pathway and collagen production through TLR4-NF-κβ pathway. *A*: alteration of inhibition capability by AHCC on HSCs activation under cytoglobin knockdown. HHSteC cells were treated with control or cytoglobin siRNA. Then HHSteC cells were treated with AHCC (500 μg/mL) and harvested at 48 h after AHCC treatment. *Left*: gene expression of activated HSCs markers of HSCs. *Right*: protein expression. Data are expressed as means ± SD (the qPCR experiment was performed in triplicate and the WB data are presented from experiments conducted with *n* = 3). **P < 0.01 (vs. vehicle). *B* and *C*: effect of TLR2 or TLR4 inhibitors on the inhibition of HSCs activation by AHCC HHSteC cells were treated with AHCC (500 μg/mL) in the presence or absence of TLR2 (50 μM) or TLR4 (5 μM) inhibitor and harvested at 48 h after AHCC treatment. *Left*: representative Western blotting images for activated HSCs markers. *Right*: quantification of Western blot bands was normalized to GAPDH. Data are presented from experiments conducted with *n* = 3.**P < 0.01 (vs. vehicle). *D* and *E*: investigation of the relationship between TLR4-NF-κβ signaling and the inhibition of collagen expression in hepatic stellate cells by AHCC. *D*: HHSteC cells were treated with TLR4 inhibitor (5 μM) for 1 h. Then HHSteC cells were stimulated by AHCC (500 μg/mL) and harvested at the indicated times. The representative Western blotting images for NF-κβ signaling are shown. *E*: HHsteC cells were treated with control or RelA siRNA for 24 h. Then HHsteC cells were treated with vehicle or AHCC for 48 h. *Righ*t: quantification of Western blot bands was normalized to GAPDH. Data are presented from experiments conducted with *n* = 3. **P < 0.01 (vs. vehicle). αSMA, α smooth muscle actin; AHCC, active hexose correlated compound; Cont, control; HHSteC, human hepatic stellate cells; HSCs, hepatic stellate cells; Inh., inhibitor; NT, no treatment; qPCR, quantitative PCR; TLR2, Toll-like receptor 2; Veh, vehicle; WB, Western blot.

We investigated whether cytoglobin induced by AHCC directly inhibits the activation of HSCs using cytoglobin siRNA. As shown in Fig. 6*A*, the suppression of *ACTA2* (αSMA) expression and the enhancement of *MMP1* expression by AHCC were attenuated by cytoglobin siRNA. However, there was no effect of cytoglobin silencing on the suppression of *COL1A1*(collagen1A) or *SERPINH1* (HSP47) expression by AHCC.

Next, we reexamined the effects of TLR2 and TLR4 inhibition on these activated HSCs markers. As expected, the addition of the TLR2 inhibitor, which is upstream of cytoglobin induction, canceled the suppression effect on αSMA expression, but it did not affect the reduction in collagen1A and HSP47 expression (Fig. 6*B*). On the other hand, the addition of TLR4 inhibitor attenuated the suppression of collagen1A expression by AHCC, but there was no change in the suppression of αSMA and HSP47 expression (Fig. 6*C*). These findings suggest that AHCC suppresses collagen1A expression via TLR4. Next, we analyzed the NF-κβ pathway, which is downstream of TLR4 (28). One of the important molecules in the NF-κβ pathway, p65, was phosphorylated upon the addition of AHCC, but this phosphorylation was reduced by the TLR4 inhibitor. Furthermore, the knockdown of p65 diminished the inhibitory effect of AHCC on collagen1A production (Fig. 6*E*). These findings suggested that the regulation of collagen1A expression in HSCs involves the NF-κβ pathway, and AHCC reduces collagen1A expression by activating NF-κβ through TLR4.

## DISCUSSION

This study demonstrated that AHCC inhibited HSCs activation by inducing cytoglobin through the TLR2-SAPK/JNK pathway and suppressing collagen production via the TLR4-NF-κβ pathway, resulting in the prevention of liver fibrosis progression. Both TLR2 and TLR4 are important for innate immunity as pattern recognition receptors that respond to pathogenesis (29). The activation of the TLR2 or TLR4 pathway during inflammation has two aspects: proinflammatory and anti-inflammatory. Stimulation of TLR2 in B-cells, macrophages, regulatory T-cells, and Kupffer cells has been reported to exert anti-inflammatory effects through the production of the anti-inflammatory cytokine IL-10, contributing to the resolution of inflammation (30, 31). On the other hand, there is also an aspect that enhances the production of proinflammatory cytokines such as TNF-α and IL-1β, worsening liver fibrosis (32, 33). Similarly, both proinflammatory and anti-inflammatory responses have been reported for TLR4 (34). At the plasma membrane, lipopolysaccharide on Gram-negative bacteria-bound TLR4 recruits the signaling adaptor MyD88 to initiate signaling and induce proinflammatory cytokines (35). In contrast, exopolysaccharide, which affects macrophages and dendritic cells in a TLR4-dependent manner, induces the expression of the inhibitory molecule IDO in bone marrow-derived dendritic cells, leading to the inhibition of T-cell proliferation (36). In this study, the expression of IL-1β and TNF-α genes in the liver showed no significant difference between the control group and the AHCC group. This suggests that AHCC has a limited proinflammatory enhancement effect mediated by TLR2 and TLR4 stimulation.

Cytoglobin was identified by Kawada et al. (16) as one of the proteins showing differential expression between the quiescent and activated states in rat HSC. So far, FGF2, hypoxia-inducing factor 1α, calcineurin, NFAT, and/or AP-1 have been reported as regulators of cytoglobin expression (25, 37). Cytocglobin is a type of globin, similar to hemoglobin and myoglobin, and has the ability to bind oxygen (38). Thus, the primary function of cytoglobin is the regulation of O_2_ homeostasis. Yoshizato et al. (39) suggested that the absence of cytoglobin disturbs O_2_ homeostasis in HSCs and increases the frequency of the generation of hypoxia. Accordingly, mitochondria become active and generate more ROS. Indeed, HSCs from cytoglobin knockout mice had higher ROS content compared with those from wild-type mice (20). Therefore, it is suggested that cytoglobin inhibits the activation of HSCs by removing ROS, which are important activators of HSCs (39). Kawada et al. (21) reported that HSCs with forced expression of cytoglobin showed reduced expression of αSMA, which is consistent with our results. From the perspective of antifibrotic effects, it is important that AHCC increased the expression of MMP-1 through the induction of cytoglobin expression. MMP-1 is a collagenase that degrade produced collagen, and the sustained inhibition of MMP-1 production by TIMP is a crucial mechanism in the pathogenesis of liver fibrosis (4). In this in vivo study, it is expected that the preventive effect of AHCC on liver fibrosis promotion is partly due to the enhancement of MMP-1 expression. Similarly, in past reports (25), it was observed that FGF2 induced the expression of MMP-1 along with cytoglobin. It is suggested that first cytoglobin was induced by FGF2, which subsequently resulted in increased expression of MMP-1. There are reports in the literature indicating that overexpression of cytoglobin in fibroblasts results in decreased expression of *TGFB1* genes (40), which is the most important cytokine in fibrosis. MMP-1 expression is suppressed by TGF-β, whereas the expression of αSMA is promoted in human fibroblast (41). In addition, the primary source of TGF-β during hepatitis is activated HSCs, and the autocrine effect further promotes the activation of the HSCs themselves (42). Therefore, cytoglobin may control the expression of MMP-1 and αSMA through the suppression of TGFβ expression.

So far, it has been reported that HSCs derived from cytoglobin knockout mice show enhanced collagen production, whereas collagen expression in HSCs derived from mice with HSC-specific forced expression of cytoglobin was lower compared with wild-type mice (21, 43). Therefore, we initially considered the possibility that cytoglobin also regulates collagen production. However, our study revealed that the reduction in collagen production induced by AHCC is not caused by the direct involvement of cytoglobin. Because of differences in the transcriptional regulation of cytoglobin between mice and humans (44), it is necessary to consider these differences when interpreting the results of our study using human hepatic stellate cell lines. It has been reported that in skin fibroblasts and gingival fibroblasts, activation of the NF-κβ pathway suppresses collagen production (45, 46). We are the first to discover a similar effect in HSCs. Brenner et al. (47) reported that NF-κβ suppresses collagen production through the Sp1 binding site in the collagen gene promoter, and our results may involve a similar mechanism. Similar to TLR2, TLR4 is also expressed in immune cells such as hepatocytes, Kupffer cells, and dendritic cells (48). Therefore, it is necessary to analyze the effects of AHCC on these TLR4s and confirm its overall impact on the liver.

NO produced from HSCs increases the production of ROS by inhibiting cytochrome c oxidase activity in mitochondria within surrounding liver cells. In addition, it has been observed that cytoglobin-deficient HSCs produce more NO during liver damage compared with wild-type HSCs (22). Based on the above, the increased production of cytoglobin induced by AHCC intake mitigates oxidative damage to surrounding liver cells. Moreover, AHCC has been reported to exhibit various hepatoprotective effects. Under IL-1β stimulation, AHCC suppresses the expression of the iNOS gene in hepatocytes, exerting an anti-inflammatory effect by reducing nitric oxide (NO) production and protecting liver cells (49) and increases hepatocyte activity by inhibiting ornithine decarboxylase induced by oxidative stress (50). In clinical trials, it has been reported that the increase in ALT levels induced by alcohol consumption significantly decreased in the AHCC intake group compared with the placebo group (51). In our study, the suppression of the increase in serum ALT levels induced by CCl_4_ administration was observed. In addition, the expression of 8-OHdG and caspase-3 was reduced by AHCC treatment, suggesting that AHCC may have directly reduced hepatocyte damage. Therefore, the inhibitory effect of AHCC on the progression of liver fibrosis is considered to be due not only to the enhancement of cytoglobin production and reduction of collagen production in HSCs but also to the reduction of the release of HSC activators through the decrease of hepatocytes damage.

AHCC is reported to have some preventive effect on liver cancer recurrence (14, 52). The involvement of cytoglobin in suppressing carcinogenesis through the reduction of cellular damage has been suggested (53–55). The cytoglobin knockout mice has been shown to spontaneously develop multiorgan cancer with aging and exhibit increased susceptibility to carcinogenesis in a liver cancer model (19, 20, 43). Considering these findings, it is expected that cytoglobin plays a role in the anticancer effects of AHCC.

Generally, polysaccharides found in mushrooms are β-glucans. Because of their large molecular weight, β-glucans are hardly absorbed from the intestines into the body. Their reported immunostimulatory effects primarily result from their action on the intestinal mucosal epithelium (56). On the other hand, the main component of AHCC is partially acylated α-glucan with a mean molecular weight of around 5,000 Daltons, and it has been reported to be absorbed from the intestinal mucosal epithelium into the body (57). α-Glucan has been reported to activate TLR2 (58) and TLR4 (59, 60) in macrophages and dendritic cells, so the bioactive substance that led to the results of this experiment may be α-glucan. Although adenosine and other bioactive substances were reported in AHCC (61), not all components have been fully elucidated yet. It is anticipated that these components or unknown components are involved in the mechanism by which AHCC suppresses HSP47 expression, which was not elucidated in this study. AHCC, which contains various active components and exerts antifibrotic effects through a combination of multiple mechanisms of action, is considered to have an advantage over drugs composed of single compounds. However, the response to TLR2 or TLR4 activation varies among different cell types such as hepatocytes, HSCs, sinusoidal endothelial cells, Kupffer cells, bile duct epithelial cells, and liver dendritic cells (62). It is necessary to further analyze the detailed effects of AHCC on TLR2 or TLR4 in each cell type using various pathological models in the future.

## DATA AVAILABILITY

Data will be made available upon reasonable request.

## GRANTS

This work was supported by Japan Society for the Promotion of Science London (JSPS) KAKENHI Grant Numbers JP17K15561, 20K16226 (to H.U.), and 21H02626 (to T.M.).

## DISCLOSURES

AHCC is a trademark of Amino Up Co., Ltd., Sapporo, Japan. H. Urushima received a research grant from Amino Up Co., Ltd. None of the other authors has any conflicts of interest, financial or otherwise, to disclose.

## AUTHOR CONTRIBUTIONS

H.U., Y.I., H.Y., and K.I. conceived and designed research; H.U., G.Q., A.D., M.T., C.K., H.N., Y.I., and H.Y. performed experiments; H.U., T.M., Y.I., and H.Y. analyzed data; H.U., T.M., Y.I., H.Y., and K.I. interpreted results of experiments; H.U. prepared figures; H.U. drafted manuscript; H.U. and K.I. edited and revised manuscript; H.U., T.M., G.Q., A.D., M.T., C.K., H.N., Y.I., H.Y., and K.I. approved final version of manuscript.
